# Shape-programmable liquid crystal elastomer structures with arbitrary three-dimensional director fields and geometries

**DOI:** 10.1038/s41467-021-26136-8

**Published:** 2021-10-12

**Authors:** Yubing Guo, Jiachen Zhang, Wenqi Hu, Muhammad Turab Ali Khan, Metin Sitti

**Affiliations:** 1 Physical Intelligence Department, Max Planck Institute for Intelligent Systems, 70569 Stuttgart, Germany; 2 Institute of Engineering Medicine, Beijing Institute of Technology, 100081 Beijing, China; 3 Department of Biomedical Engineering, City University of Hong Kong, Hong Kong SAR, China; 4 Institute for Biomedical Engineering, ETH Zurich, 8092 Zurich, Switzerland; 5 School of Medicine and College of Engineering, Koç University, 34450 Istanbul, Turkey

**Keywords:** Actuators, Liquid crystals

## Abstract

Liquid crystal elastomers exhibit large reversible strain and programmable shape transformations, enabling various applications in soft robotics, dynamic optics, and programmable origami and kirigami. The morphing modes of these materials depend on both their geometries and director fields. In two dimensions, a pixel-by-pixel design has been accomplished to attain more flexibility over the spatial resolution of the liquid crystal response. Here we generalize this idea in two steps. First, we create independent, cubic light-responsive voxels, each with a predefined director field orientation. Second, these voxels are in turn assembled to form lines, grids, or skeletal structures that would be rather difficult to obtain from an initially connected material sample. In this way, the orientation of the director fields can be made to vary at voxel resolution to allow for programmable optically- or thermally-triggered anisotropic or heterogeneous material responses and morphology changes in three dimensions that would be impossible or hard to implement otherwise.

## Abstract (web summary)

Pre-patterning director fields into liquid crystal elastomers enables programmed morphing upon actuation. Guo et al. realize 3D skeletal assemblies with previously non-achievable director fields and morphing modes.

## Introduction

Programmable shape-morphing of stimuli-responsive materials is vital for the functionalities of smart material-based devices in various applications, such as robot locomotion^1–3^, targeted drug delivery^4–6^, and tunable surface wettability^7–9^. Liquid crystal elastomers (LCEs) exhibit programmable complex shape transformations, depending on both the programmed geometries and molecular orientations (i.e., the director fields)^10–14^. Programming the geometries and the director fields of LCEs have thus been studied and utilized as an effective approach to design the shape-morphing behavior of LCEs. For example, Aharoni et al. demonstrated arbitrary surface geometries transformed from a thin LCE film by inversely designing arbitrary two-dimensional (2D) LCE director fields^15^. Further improvement in LCE-based shape-morphing, especially in the third dimension, would trigger traditionally non-achievable morphing modes, such as morphing between two arbitrary three-dimensional (3D) shapes. However, this improvement requires freely encoding the LCE’s arbitrary 3D geometries and arbitrary 3D director fields, which is not possible yet.

Conventionally, thin and flat LCE films are fabricated with either uniform or nonuniform director fields introduced by mechanical stretching^16^, confining surfaces^17^, and external magnetic field^18^. Various fabrication techniques have recently been developed to pattern liquid crystals (LCs) into arbitrary 2D in-plane director fields, enabling complex 2D to 3D shape transformation. These existing techniques include nano-rubbing with atomic force microscope (AFM) tips^19^, digital micro-mirror device-based photopatterning^20^, plasmonic photopatterning^21–23^, pixel-by-pixel photopatterning^11,24^, micro-channels from direct laser writing^12,25^, and 3D printing of dynamic networks^26^. Based on these techniques, various LCE-based stimuli-responsive shape-morphing has been demonstrated, e.g., iris-like device and human face from a flat film^15,27^.

Recent advances in 3D printing technologies have enabled the creation of 3D LCE geometries via ink^28–32^ and optical 3D printing^10,13,33,34^, which allows for 3D-to-3D shape-morphing. Ink 3D printing features a millimeter-sized structure. Additionally, the printed LCE director field is along with the extruded fiber and cannot change abruptly without changing the printing path. At last, the current extrudable LCE materials do not have a high enough elastic modulus to sustain a printing path along the *z* direction^31^. Optical 3D printing features a high resolution up to hundreds of nanometers and complex LCE director fields^34^. Especially, Tabrizi et al. demonstrated 3D LCE geometries with arbitrary 2D in-plane director fields by combining optical 3D printing and rotatable permanent magnets^13^. Guo et al. also reported a two-photon polymerization technique between two surface-patterned confining glasses^10^. It enables arbitrary 2D director fields ${{{{{\boldsymbol{n}}}}}}(x,y,0)$ with a maximum height limitation of 100 μm. However, the fabrication of LCEs with both arbitrary 3D geometries and arbitrary 3D director fields is still challenging and in high demand for enriching desirable 3D-to-3D morphing modes towards real-world applications.

Here, we report an approach to fabricate LCEs with uncoupled programmable arbitrary 3D geometries and arbitrary 3D director fields by 3D-assembling microscale heterogeneous LCE voxel building blocks, which are either 60 or 100 µm side-length cubes with arbitrarily selected uniform director fields ${{{{{\boldsymbol{n}}}}}}(x,{{{{{\rm{y}}}}}},{{{{{\rm{z}}}}}})$ fabricated from two-photon polymerization. This work programs arbitrary 3D director fields ${{{{{\boldsymbol{n}}}}}}(x,{{{{{\rm{y}}}}}},{{{{{\rm{z}}}}}})$ on arbitrary 3D bodies of LCEs. Specifically, we present one-dimensional (1D), 2D, and 3D structures with traditionally non-achievable director fields and demonstrate exotic morphing modes enabled by the nonzero *z*-component of the programmed director fields. In addition, due to the anisotropic mechanical, thermal, electrical, and optical properties of LCEs along and perpendicular to the local director field, arbitrary 3D initial shapes and director fields would enable 3D LCE devices with heterogeneous physical properties.

## Results

### Design of individual LCE voxels with arbitrary 3D director fields

**Fig. 1:**
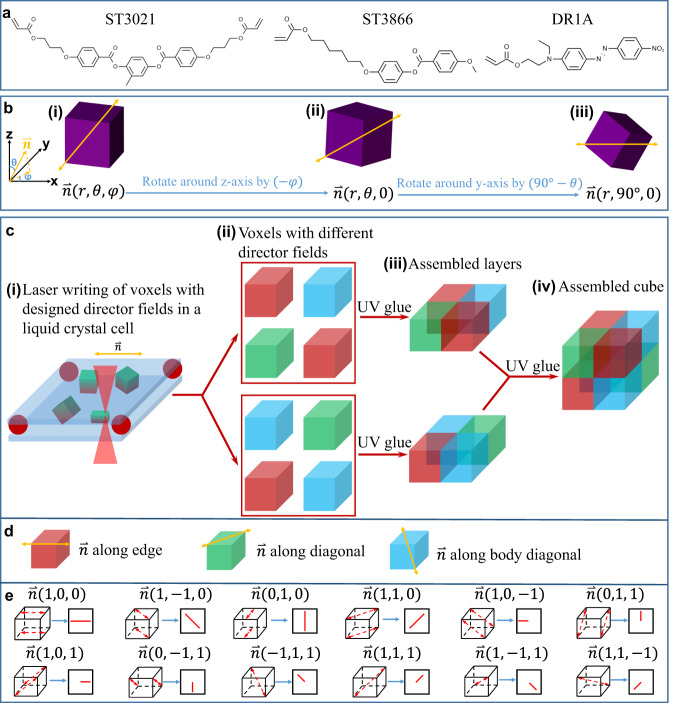
Schematic of the fabrication process. The 3D liquid crystal elastomer (LCE) structures are fabricated by a 3D assembly of heterogeneous microscale LCE voxels with programmed director fields. **a** Chemical structures of the utilized liquid crystal (LC) monomers ST3021, ST3866, and a dye DR1A (disperse red 1 acrylate). **b** A voxel with a director field along the *x*-axis is achieved by a two-stage rotation of a voxel with an arbitrary director field. **c** Schematic fabrication process of single voxels and their 3D-assembly process. **d** Three representative voxels with director fields along the edge, surface diagonal, and body diagonal, respectively. **e** 3D director fields expressed with 2D images.

The schematic of the fabrication process of the 3D-assembled LCE voxels with programmed director fields is illustrated in Fig. 1. Creating the individual LCE voxels with programmable director fields ${{{{{\boldsymbol{n}}}}}}(x,{y},{z})$ is the key step in such a process. The LC material used is a mixture of 4-methoxybenzoic acid 4-(6-acryloyloxyhexyloxy) phenyl ester (ST3866, LC monomer), 1,4-Bis[4-(3-acryloyloxypropyloxy)benzoyloxy]-2-methylbenzene (ST3021, crosslinker), disperse red 1 acrylate (dye), and Irgacure 369 (photoinitiator). The chemical structures of these materials can be found in Fig. 1a. Mixtures of these materials were filled into LC cells with a 150 μm cell gap. In these LC cells, cube-shaped voxels were later fabricated by a two-photon polymerization process.

In Fig. 1b, the arbitrarily selected uniform director field of a voxel is described as ${{{{{\boldsymbol{n}}}}}}(x,{y},{z})$, which can be expressed as ${{{{{\boldsymbol{n}}}}}}(r,\theta ,\varphi )$ in a spherical coordinate system (Fig. 1b(i)), where *r* is the radial distance, ${{{{{\rm{\varphi }}}}}}$ is the azimuthal angle, and ${{{{{\rm{\theta }}}}}}$ is the polar angle. To fabricate this voxel, we first assembled an LC cell within which we later two-photon polymerizate the voxel (Fig. 1c and Methods). This LC cell has a director field along the *x*-axis that corresponds to a predefined director field ${{{{{{\boldsymbol{n}}}}}}}_{{{{{{\boldsymbol{p}}}}}}}(r,\ 90^{{\circ }},0)$. As shown in Fig. 1b(i–iii), any arbitrary 3D director field $\,{{{{{{\boldsymbol{n}}}}}}}_{{{{{{\boldsymbol{a}}}}}}}(r,\,\theta ,\varphi )$ can be transferred into ${{{{{{\boldsymbol{n}}}}}}}_{{{{{{\boldsymbol{p}}}}}}}(r,\ 90^{{\circ }},0)$ via the following two-step rotation: (1) ${{{{{{\boldsymbol{n}}}}}}}_{{{{{{\boldsymbol{a}}}}}}}(r,\theta ,\varphi )$ rotates around the *z*-axis by $-{{{{{\rm{\varphi }}}}}}$ and becomes ${{{{{{\boldsymbol{n}}}}}}}_{{{{{{\boldsymbol{t}}}}}}}(r,\,\theta ,0)$; (2) ${{{{{{\boldsymbol{n}}}}}}}_{{{{{{\boldsymbol{t}}}}}}}(r,\ \theta ,0)$ rotates around *y*-axis by ${{\theta }}{{{{{\rm{-}}}}}}90^{{\circ }}$ and becomes ${{{{{{\boldsymbol{n}}}}}}}_{{{{{{\boldsymbol{p}}}}}}}(r,\ 90^\circ ,0)$. Therefore, as schematically shown in Fig. 1b, rotating the voxel together with the director field ${{{{{{\boldsymbol{n}}}}}}}_{{{{{{\boldsymbol{a}}}}}}}(r,\theta ,\varphi )$ leads to a new voxel after this two-stage rotation. Then, by polymerizing the rotated voxel, shown in Fig. 1b(iii) and Fig. 1c(i), with two-photon 3D polymerization inside the LC cells, we obtained a voxel with the desired director field ${{{{{{\boldsymbol{n}}}}}}}_{{{{{{\boldsymbol{a}}}}}}}(r,\theta ,\varphi )$. In principle, ${{{{{\rm{\theta }}}}}}$ can vary from 0 to $180^\circ$, and ${{{{{\rm{\varphi }}}}}}$ can change from 0 to $360^\circ$. For convenience, the rest of this manuscript uses a Cartesian coordinate system, in which ${{{{{{\boldsymbol{n}}}}}}}_{{{{{{\boldsymbol{a}}}}}}}(r,\theta ,\varphi )$ can be expressed as ***n***_***a***_(rsinθ cosφ, rsinθ sinφ, rcosθ).

Finally, these voxels with arbitrary director fields are 3D-assembled together (Fig. 1c(iii–iv)) to form arbitrary geometries with any desired heterogeneous profiles of the director field ${{{{{\boldsymbol{n}}}}}}(x,{{{{{\rm{y}}}}}},{{{{{\rm{z}}}}}})$. In contrast, previous programmable LC alignment is limited to 2D, which is arbitrary in-plane director fields ${{{{{\boldsymbol{n}}}}}}(x,\ {{{{{\rm{y}}}}}},0)$ with zero *z*-component. Director fields with nonzero *z*-component are only realized either by combining in-plane surface alignment and external electric field^35^ or by hybrid in-plane and vertical surface alignments^27^. Both strategies only allow for very limited continuous changes of the *z*-component.

For simplicity, we focused on using LCE voxels with three representative uniform director fields (Fig. 1d) in this work: one along the edge ${{{{{\boldsymbol{n}}}}}}(1,\ 0,0)$, another along surface diagonal ${{{{{\boldsymbol{n}}}}}}(1,\ 1,0)$, and the last one along body diagonal ${{{{{\boldsymbol{n}}}}}}(-1,1,1)$. Notice that by simply switching their top, front, and right surfaces, these three voxels can be transferred into different ones. For example, a voxel with ${{{{{\boldsymbol{n}}}}}}(1,\ 0,0)$ can be transferred into one with ${{{{{\boldsymbol{n}}}}}}(0,\ 1,0)$ and also one with ${{{{{\boldsymbol{n}}}}}}(0,\ 0,1)$. For convenience, we represent these 3D director fields with 2D images, as shown in Fig. 1e.

### Characterization of the fabricated LCE voxels

**Fig. 2:**
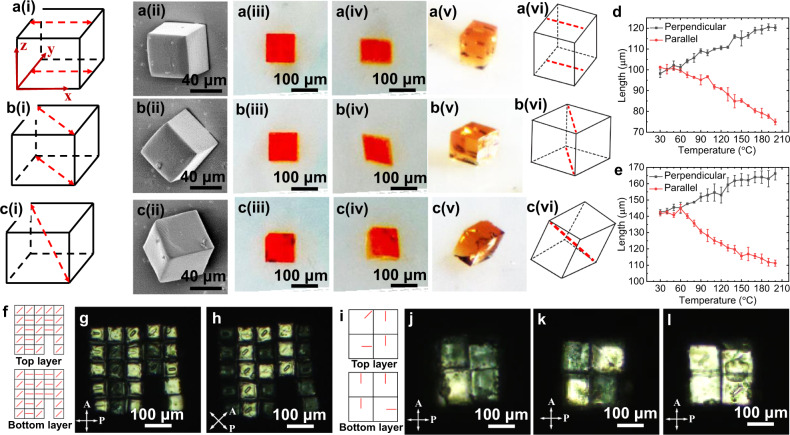
Characterization of the individual LCE voxels and the 3D-assembled LCE structures. **a**–**c** Characterization of individual voxels with director fields along with the edge (**a**), along the surface diagonal (**b**), and along the body diagonal (**c**); (i)–(vi) include schematics of the programmed director fields (i), SEM images of the fabricated voxels (ii), photos of the fabricated voxels at 30 °C (iii), photos of the voxels actuated at 150 °C (iv), 3D-view photos of the actuated voxels (v), and schematics of the actuated voxels (vi). **d** Length of two edges parallel and perpendicular to the director field as a function of temperature for the voxel in (**a**). **e** Length of two diagonals parallel and perpendicular to the director field as a function of temperature for the voxel in (**b**). The error bars in (**d**) and (**e**) indicate the standard deviation of the measured length. **f** Schematic director field of the top and the bottom layers. **g** A letter “M” is shown under a polarized optical microscope (POM). **h** A letter “P” is shown when the sample in (**g**) is rotated by 45° under POM. **i** Schematic director field of a cube. **j–l** POM images of an assembled cube viewed from the top surface (**j**), the front surface (**k**), and the right surface (**l**). Note that we define the two surfaces perpendicular to the *z*-axis as top and bottom surfaces, the two surfaces perpendicular to the *y*-axis as front and back surfaces, and the two surfaces perpendicular to the *x*-axis as right and left surfaces.

We fabricated the above-mentioned three kinds of representative voxels via two-photon polymerization. Their corresponding azimuthal angle *φ* and polar angle *θ* are presented in Supplementary Fig. 1, which were used to design the two-stage rotation for each kind of voxels. We fabricated cubic voxels with two different sizes, one with 100 μm edge-length and the other with 60 μm edge-length. First, we characterized the single voxels with different director fields. The columns in Fig. 2a–c present schematic drawings (i), scanning electron microscope (SEM) images (ii), bright-field optical microscope images of the fabricated voxels (iii), bright-field images (iv), 3D-view bright-field images (v), and schematic drawings (vi) of the actuated voxels at high temperature. The SEM images exhibit a nearly perfect cubic shape for all three kinds of voxels. By comparing images in (iii) and (iv), we observed that a voxel expanded and shrunk along the direction perpendicular to and parallel with its director field, respectively. We measured these expansion and shrinkage values as a function of temperature for voxels in Fig. 2a, b and presented the results in Fig. 2d, e. For the first voxel, strains along and perpendicular to the director fields were around −0.25 and 0.20; and for the second voxel, strains were around −0.22 and 0.18 along two directions.

One interesting property of these voxels is that they show different brightness when observed from different surfaces (Supplementary Fig. 2) under a polarized optical microscope (POM). For example, the voxel with the director field along the surface diagonal is dark when observed from the front (*x*-*z* plane) and right surfaces (*y*-*z* plane), while bright when observed from the top surface (*x*-*y* plane). In Supplementary Fig. 2, we also characterized the shape transformation of these voxels with different director fields by dipping them into dimethylformamide (DMF), which indicated that these voxels contract along the director field and expand perpendicular to it.

### 3D-assembly of the LCE voxels for creating arbitrary 3D geometries

Due to the versatility of the employed 3D-assembly approach, almost any 3D geometries can be obtained by heterogeneously assembling LCE voxels. As schematically shown in Fig. 1c, we can first assemble LCE voxels into different layers and then assemble these layers into 3D structures. Each voxel was placed at the designated location with a prespecified orientation within the 3D overall structure of the device. An optical adhesive (NOA60, Norland) was applied at the interface between adjacent voxels, then cured under ultraviolet (UV) light exposure (365 nm, 1 J/cm^2^), and finally pulled discrete voxels together into a 3D continuum.

Optical characterization is a simple yet effective method to verify both geometries and director fields of the assembled LCE prototypes. In order to show the capability of fabricating LCEs with programmable 3D geometries and 3D director fields, we designed and assembled two LCE structures with encoded geometries and director fields and presented their POM images with programmed properties. First, we built a bilayer structure using the two different kinds of voxels (${{{{{\boldsymbol{n}}}}}}(1,0,0)$ and ${{{{{\boldsymbol{n}}}}}}(1,1,0)$), which displayed different brightness when they were rotated by $0^\circ$ and by $45^\circ$, as shown in Supplementary Fig. 2. We fabricated a two-layer 46-voxel LCE structure, showing the letter “M” when the rotation is $0^\circ$ while switching to the letter “P” when the rotation is $45^\circ$. The 23 voxels in two (bottom and top) layers can be categorized into three types: type 1: only bright at $0^\circ$; type 2: only bright at $45^\circ$; and type 3: bright at both angles. Each type of voxel can be realized by stacking different voxels on the top and the bottom layer. Specifically, two voxels with ${{{{{\boldsymbol{n}}}}}}(1,1,0)$ director field correspond to type 1, two voxels with ${{{{{\boldsymbol{n}}}}}}(1,0,0)$ director fields correspond to type 2, and top voxel with ${{{{{\boldsymbol{n}}}}}}(1,1,0)$ and bottom voxel with ${{{{{\boldsymbol{n}}}}}}(1,0,0)$ director fields correspond to type 3. Note that we were able to leave two empty voxels in both layers that are dark at both angles, which is another advantage of the versatile assembly approach. A detailed director field design is presented in Fig. 2f. The experimental results are shown in Fig. 2g, h, agreeing with our design.

Another optical demonstration is presented in Fig. 2i–l with a (2 × 2 × 2)8-voxel cube, which shows three different images when viewed from three different perspectives under POM. This effect is designed according to different POM images of single voxels when viewed from different perspectives (Supplementary Fig. 2). The eight voxels used to fabricate the above cube have encoded director fields shown in Fig. 2i. The POM images observed from different perspectives (top surface, front surface, and right surface corresponds to Fig. 2j–l, respectively) agree with these encoded voxel director fields.

From the above two examples, we demonstrate the capability of the reported approach to creating 3D LCE structures with programmable director fields. While these examples do show heterogeneous optical properties, the glue used currently may create light scattering issues at the interface. Therefore, these demonstrations are primarily intended to verify that the assembled LCE structures possess the designed 3D shapes and director fields. The UV glue is used since it is optically clear, in contrast to the translucent unpolymerized LCE. This eases the pick-and-place manipulation by the operator. This scattering issue can be resolved in the future by using the uncured LCE itself as the glue with automatic equipment to apply it precisely^36^.

### Reversible shape transformation of the 3D LCE structures

Programmable complex reversible shape transformation is one characteristic advantage of LCEs compared with other stimuli-responsive materials^11,14^. The achievable morphing modes depend on both the geometry and the director fields of the LCE structure. Therefore, our strategy of fabricating LCE structures with both arbitrary geometries and arbitrary director fields enables various shape-morphing modes. In the following sections, we designed and demonstrated 1D, 2D, and 3D structures with previously non-achievable director fields and studied their shape transformation behaviors.

**Fig. 3:**
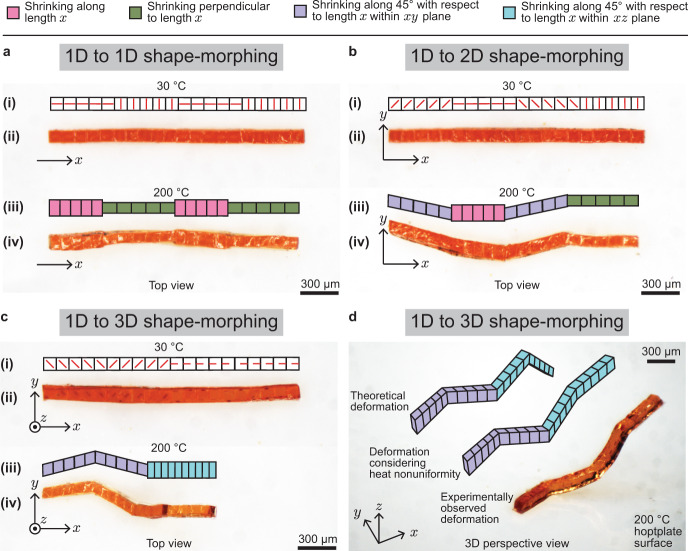
Thermal 1D-to-1D/2D/3D shape transformation of the cubic voxels assembled in 1D lines. **a**–**c** Assembled 1D lines transforming into a 1D line with a varying width (**a**), a 2D line (**b**), and a 3D line (**c**), where (i, ii) include the schematic director fields and photos of the non-actuated 1D lines at 30 °C, and (iii, iv) include the schematic drawing and photo of the actuated 1D lines at 200 °C. **d** 3D detailed schematic views and 3D-view photo of the 1D line transforming into a 3D line at 200 °C in (**c**).

We started with assembled 1D lines with voxels encoded with designed director fields. Previously, 1D lines were usually fabricated with a uniform director field along their length^37,38^. In contrast, the reported approach assembles the LCE voxels to form 1D lines with an arbitrary profile of 3D director fields. Here, we exhibited LCE lines with three different encoded profiles of director fields, as shown in Fig. 3. Visually similar lines with 100 µm width, 100 µm height, and 2 mm length transformed into dramatically different shapes after being activated by heating. First, Fig. 3a shows a 1D line with four sections. After being activated, sections 1 and 3, counting from left, expand transversely while sections 2 and 4 expand longitudinally (Fig. 3a–iii, iv). Second, Fig. 3b shows a line with four sections. After being activated, sections 1 and 3 deform obliquely in the *x-y* plane. Section 2 deforms transversely and section 4 deforms longitudinally. Third, Fig. 3c shows a line with four sections. After being activated, the section 1 and 2 deform in the *x-y* plane. Section 3 and 4 deform in the *x-z* plane. Figure 3d shows the result in Fig. 3c from another perspective, considering the heating nonuniformity. In contrast, the ink 3D printing^28–32^ can print a 1D line, but the director field is constrained to be along its printing path^31^. Also, the optical 3D printing^10,13,33,34^ techniques can potentially manufacture 1D lines between two substrates^10^ (with a gap distance less than 100 µm), allowing arbitrary 2D director fields ${{{{{\boldsymbol{n}}}}}}(x,y,0)$.

**Fig. 4:**
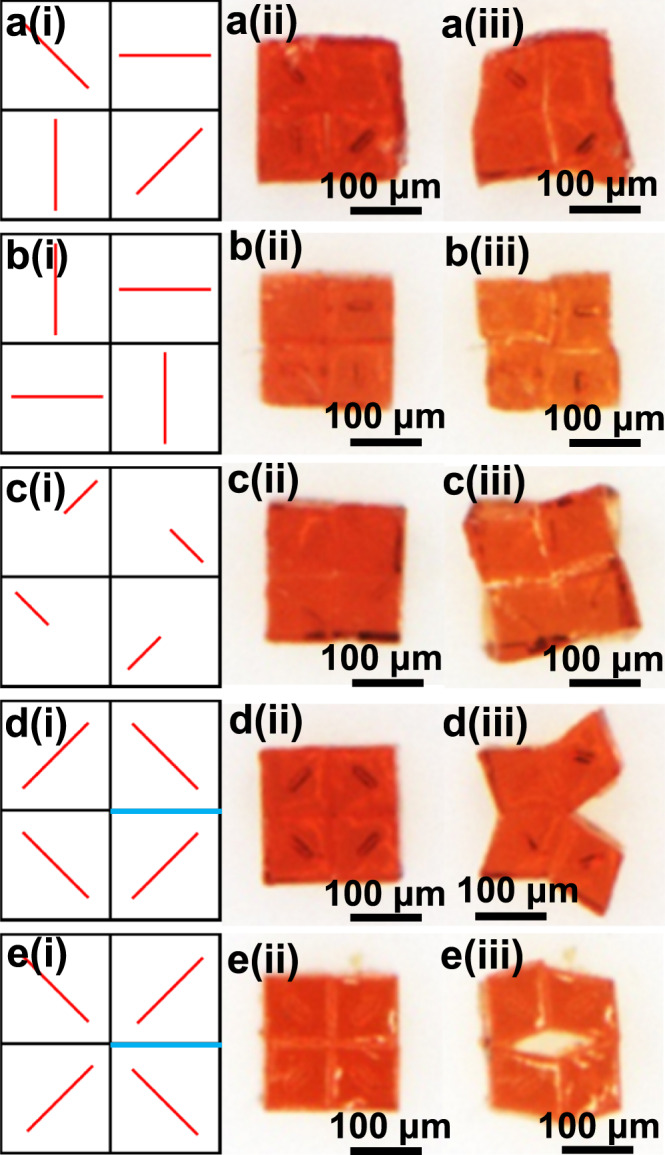
Thermal 2D-to-2D shape transformation of the assembled different 2D LCE structures. Assembled 2D LCE structures **a**–**c** with all surfaces glued together and **d**, **e** with a non-glued surface (marked with the blue line), transforming into different shapes. (i) Schematic of the programmed director fields. (ii) Top-view photos of the 2D LCE structures at 30 °C. (iii) Actuated, shape-morphed 2D structures at 200 °C.

We then fabricated 2D LCE structures with encoded 3D director fields. Previously, LCE films with arbitrary 2D in-plane director fields ${{{{{\boldsymbol{n}}}}}}({{{{{\rm{x}}}}}},{{{{{\rm{y}}}}}},0)$ have been realized via strategies such as rotating permanent magnet^13^, plasmonic photopatterning^21^, pixel-by-pixel laser writing^11^, and micro-rubbing with AFM tips^19^. In contrast, here we demonstrated 2D structures with arbitrary 3D profiles of director fields, especially with a nonzero *z*-component director field. We presented five cases with four voxels and designed director fields in Fig. 4. We observed from Fig. 2 that, when these voxels are actuated with high temperature, voxels with ${{{{{\boldsymbol{n}}}}}}(1,1,0)$ director field would change their angles: the two angles become larger along the director field, while the two angles become smaller perpendicular to the director field. As a result, the structure in Fig. 4a with an initial square 2D shape transformed into a kite shape after actuation. In contrast, the other two kinds of voxels (${{{{{\boldsymbol{n}}}}}}\left(1,0,0\right)$ and ${{{{{\boldsymbol{n}}}}}}(1,1,1)$) show much smaller changes of angles. The structures in Fig. 4b, c formed spiral lines after actuation due to shrinkage and expansion on two sides of each line. Interestingly, four angles at the center all became smaller (larger) for the structures in Fig. 4d (Fig. 4e). In these two cases, if we had thin enough film, they would be transformed into 3D structures. However, the film thickness is comparable with the film size in the current case. Therefore, we left one edge in each case un-bonded, which are marked with blue lines in Fig. 4d, e. Upon thermal actuation, they transformed into different 2D structures.

In these examples, we exhibited several advantages of the proposed strategy: we can choose some un-bonded parts in the assembled structures to enrich morphing modes; we demonstrate 2D LCE structures with nonzero *z*-component programmable director fields ${{{{{\boldsymbol{n}}}}}}(x,{y},{z})$, which is impossible with conventional methods; we exhibit morphing behaviors of LCE 2D structures with programmed director fields and large thickness and size ratio, which has been rarely explored before.

**Fig. 5:**
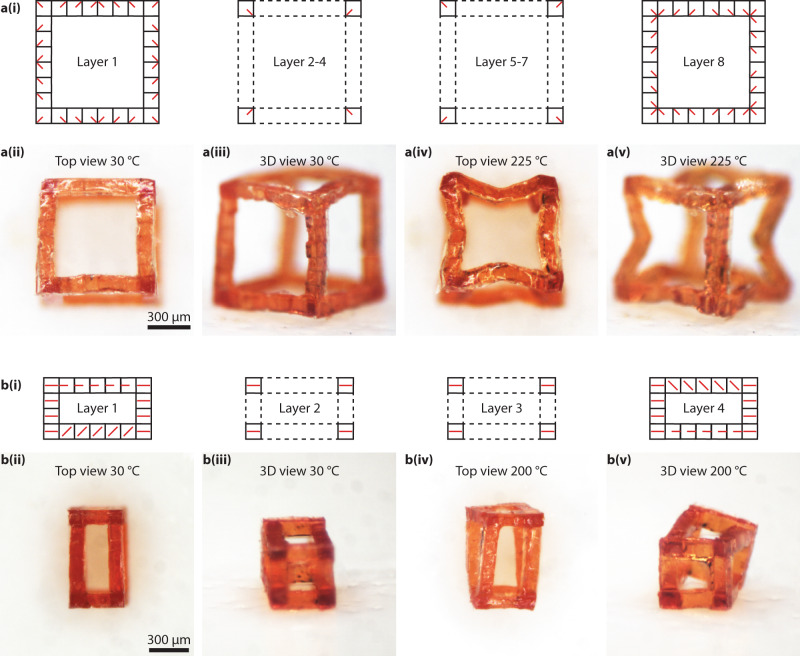
Thermal 3D-to-3D shape transformation of the assembled 3D LCE structures. **a**, **b** Thermal actuation of a cuboid LCE frame with (8-layer) 80 voxels **a** and a rectangular LCE frame with 44 voxels. (i) Schematic of the programmed director field of the cuboid frame, (ii) top-view, and (iii) 3D-view photos of the cuboid frame at 30 °C. (iv) Top-view and (v) 3D-view photos of the actuated cuboid frame at the elevated temperature of 200 °C.

Finally, we fabricated 3D LCE structures with designed 3D director fields. Due to the versatility of the reported approach, we can assemble voxels with arbitrary director fields, including those with nonzero *z*-component, to make arbitrary 3D profiles of director fields that are uncoupled from the 3D geometries. Figure 5a shows an assembled 80-voxel cube frame with each voxel possessing a director field with nonzero *z*-components (Fig. 5a(i)). While we indeed can make a structure with a larger fill factor (Supplementary Fig. 6), this design is used to enable significant deformation, similar to previous literatures^39–41^. The cube has a dimension of 800 µm^3^ × 800 µm^3^ × 800 µm^3^. Upon thermal actuation, all the edges bent towards the cube body center (Fig. 5a(ii–v)). Such a design can be potentially useful in caging micro-objects^42^. The second example in Fig. 5b contains a 36-voxel rectangular frame. The rectangular frame has a dimension of 400 µm^3^ × 700 µm^3^ × 400 µm^3^.

Figure 5b(i) shows the design of the director field and 3D geometry, where layers 1 and 4 contain voxels with nonzero *z*-components. We designed this assembled structure with the morphing modes that the top surface will rotate with respect to the bottom surface upon thermal actuation, where the top and bottom surfaces are the two surfaces parallel to the *x-z* plane, as shown in Fig. 5b(ii). To implement such a design, the director field direction in two long edges in layer 1 and layer 4 is rotationally symmetric (Fig. 5b(i)). The experimental results presented in Fig. 5b(iv) show the top and bottom surfaces are still parallel after activation, while four long edges deform obliquely and create an angular displacement between top and bottom surfaces. This observation agrees with our design. At last, we show in Supplementary Video 1 that the temperature-induced deformation is reversible for the structure shown in Fig. 5a. We also show in Supplementary Video 2 that UV illumination can cause a similar reversible deformation for the structure shown in Fig. 5b, with the help of dispersed red 1 acrylate. Since the UV lamp used was hot, the deformation in the latter case could attribute to both the heat from the environment and the UV-induced in situ heat inside the voxels.

The overall size of these structures shown in Fig. 5 is well below 1 mm. The director field direction can also be easily changed along each edge for every 100 µm, as shown in Fig. 5a(i) and b(i). Indeed, the dimension of the voxels used here (100 µm) approaches that possible with the extrusion-based 3D printing approach^28–32^. However, the extrusion methods constrain the director field to be along with the longitudinal direction of the fiber. Therefore, it is very challenging, if not impossible, to change the director field direction without changing the printing path. Practically, it means the extrusion method cannot draw each edge of the frame shown in Fig. 5, while changing the director field for every 100 µm step. The same challenge also applies to making structures demonstrated in Figs. 2f–l, 3c, and 4c, all of which are successfully demonstrated by the proposed assembly method. Finally, the 3D structures shown in Fig. 5 is already too large for optical 3D printing^10^, which takes place between two substrates with a small gap (less than 100 µm), not mentioning that achievable director fields in the optical 3D printing method have to be in-plane aligned ${{{{{\boldsymbol{n}}}}}}(x,{y},0)$.

In the experiment, we tried to use a very small amount of UV glue to minimize its impact on the shape-morphing of LCE. We observed the shape-morphing of all assembled structures in Figs. 3 and 5 agrees well with the predicted shapes, which indicates that the existence of the UV glue does not affect shape-morphing much. To further support this, we also investigated the difference between the directly printed and assembled LCE structures. The result is shown in Supplementary Fig. 7. Both vertical (Sample 1 and 2) and horizontal director fields (Sample 3 and 4) were investigated. As the experiments suggest, the difference between the assembled and printed beams is negligible. Therefore, we conclude that the effect of the glue is negligible as long as the thickness of the UV glue is much smaller than the size of the LCE voxels. To achieve future extremely precise shape transformations, especially those with complex director fields, better control of the glue layer thickness is necessary. Such requirements can be met in at least two ways. First, the assembling process of the LCE voxels can employ an assisting 3D-microprinted jig using two-photon polymerization^43^. The jig can hold two neighboring voxels at exact positions with a controlled middle gap distance, e.g., 5 µm. Then, the glue can be poured into the gap to control the glue thickness precisely, where the excess glue can be removed easily. Here, the glued structure can be removed from the jig after the assembly process and glue curing are over. Second, LCE voxels with excessive reactive acrylate groups can be fabricated by adjusting chemical reactions during the fabrication process. Then, these voxels would be assembled together without a glue layer and bonded together with permanent covalent bonds, which can be formed by the polymerization of the acrylate groups^44^.

## Discussions

Based on the design principle of the LCE voxels with programmable director fields and the versatility of the heterogeneous 3D-assembly method, we achieved assembled LCE structures with both arbitrary geometries and arbitrary director field profiles. Three main challenges exist for the reported approach. First, proof-of-concept manual assembly employed at the current stage is a serial process with relatively low throughput, requiring a longer time to fabricate larger or more complex structures with a much higher number of voxels. Second, the voxel size should be larger than $\sim$50 µm for an effective assembly, which limits the resolution of this strategy. Finally, due to the limited surface anchoring strength of the LC monomer in the LC cells, maximal voxel size is limited to 100 µm currently, which set the maximal assembled structure size to be a few millimeters considering the serial assembly process speed. Such challenges can be addressed by employing advanced automated robotic micromanipulation systems in the future^36,45–47^.

LCE structures are anisotropic, where almost all physical properties are different along with the directions parallel and perpendicular to their local director fields^14^. In this work, we demonstrated the anisotropic optical properties of the assembled LCE structures in Fig. 2 and Supplementary Fig. 2. Mechanical anisotropy was also recently demonstrated in a thin film patterned into domains with different director fields^48^. We expect that the proposed versatile approach to fabricate LCE structures with arbitrarily programmable 3D geometries and 3D director fields would enable a wide range of applications in the construction of 3D LCE devices with programmable heterogeneous physical properties.

Compared with other stimuli-responsive materials, one major advantage of the LCEs is their programmable shape transformations, which are encoded in both director fields and initial geometries. Therefore, the reported strategy for fabrication of LCEs with arbitrary 3D director fields and arbitrary 3D geometries would significantly enrich the morphing modes of LCEs and thus improve their functionalities and applications in various fields, such as soft robotics and programmable metamaterials, origami, and kirigami. In addition, we expect that 3D shape-morphing between two arbitrary shapes is possible by combining our reported strategy and proper inverse design methods^15^.

## Methods

### Materials

4-methoxybenzoic acid 4-(6-acryloyloxyhexyloxy) phenyl ester (ST3866) and 1,4-Bis[4-(3-acryloyloxypropyloxy)benzoyloxy]-2-methylbenzene (ST3021) were purchased from Synthon Chemicals. Irgacure 369 and disperse red 1 acrylate (DR1A) was purchased from Sigma Aldrich. All the materials were used as received.

### Liquid crystal (LC) cells

Glass substrates were cleaned with deionized water, acetone, and isopropanol sequentially and then were spin-coated with polyimide 2555 at a spin speed of 3000 rpm for 30 s. Then, the coated substrates were baked at 110 °C for 10 min to evaporate the solvent and 180 °C for 30 min to polymerize the polyimide. The thin polyimide layer was then rubbed unidirectionally with a cloth to achieve the uniform alignment of LCs. Two glass substrates were assembled to form an LC cell with 150 µm spacers to maintain the cell thickness. A mixture of 66 wt% ST3866, 33 wt% ST3021, and 1 wt% Irgacure 369 was filled into a cell with capillary force at 100 °C hot stage. The cell was then cooled slowly to room temperature.

### Fabrication of the LCE voxels

LCE voxels with three representative uniform director fields were fabricated with a direct laser writing system (Nanoscribe GmbH) equipped with a 25X objective. To achieve voxels with director fields along the edge, diagonal, and body diagonal of cubes, we designed three kinds of cubes, as schematically shown in Fig. 1d. These cubes were designed with SolidWorks and fabricated from two-photon polymerization. Then, the LC cell is separated, and the glass substrates are dipped in isopropanol (on an 80 °C hot stage) for 3 min to remove the unexposed photoresist.

### Assembly of the LCE voxels

The 3D-assembly process was carried out under a stereomicroscope (ZEISS Stemi 508, Carl Zeiss Microscopy GmbH) with magnification values ranging between 6.3x and 50x. The zoom and focus of the microscope could be easily adjusted by two sets of rotary knobs symmetrically located at both its left and right sides. This microscope allows the user to quickly adjust the zoom and the focus of the field of view, which is especially useful when assembling 3D structures. Besides the microscope, tweezers (5-SA Outils Rubis SA, Switzerland) or iron needles (Agani 30 G needles, Terumo) were used as the primary handheld tools to perform the assembly. A 0.03 mm diameter copper wire was nested within a Seque/Pro capillary tip (Bio-Rad Laboratories) as a tool to apply the optical adhesive at the desired location to form bonding. The aforementioned instruments are low-cost and easy to access for most people in academia and industry.

## Supplementary information

Supplementary Information

Description of Additional Supplementary Files

Supplementary Movie 1

Supplementary Movie 2

## Data Availability

All data generated or analyzed during this study are included in the published article and its Supplementary Information and are available from the corresponding author on reasonable request.

## Code availability

No code is used in this manuscript.
