# Supplementary material for: Shape-programmable liquid crystal elastomer structures with arbitrary three-dimensional director fields and geometries
Source: Nat Commun. 2021 Oct 12;12:5936. doi: 10.1038/s41467-021-26136-8 (PMC8511085; doi:10.1038/s41467-021-26136-8)
Supplement: Supplementary file 1 — Supplementary Information [file 41467_2021_26136_MOESM1_ESM.pdf]

Supporting Information

Shape-Programmable Liquid Crystal Elastomer Structures with Arbitrary Three-Dimensional Director Fields and Geometries

Yubing Guo†, Jiachen Zhang†, Wenqi Hu†, Muhammad Turab Ali Khan, and Metin Sitti*

† Equally contributing first authors
* Correspondence to: sitti@is.mpg.de

Supplementary Figures

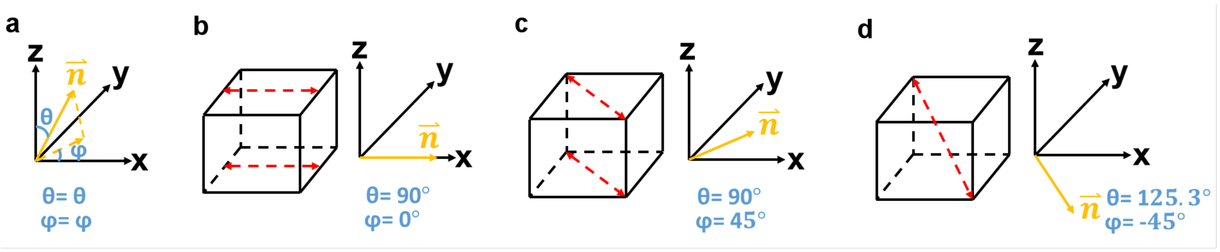

Supplementary Figure 1. **Three representative voxels in the spherical coordinate system.** a) An arbitrary director field in a spherical coordinate system. b-d) Azimuthal and polar angles for three representative director fields: along the edge (b), along the surface diagonal (c), and along the body diagonal (d).

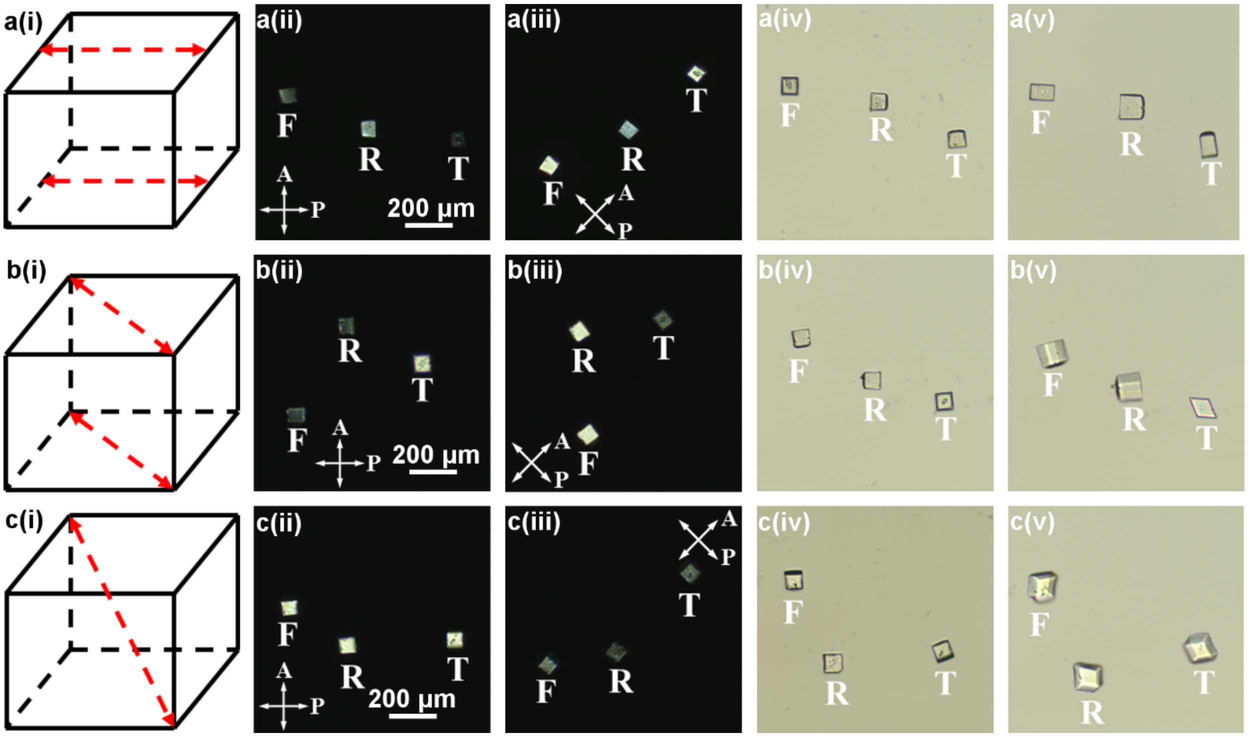

Supplementary Figure 2. **Characterization of the individual voxels with different director fields.** a-c) Polarized optical microscope (POM) characterization and N,N-Dimethylformamide (DMF) swelling of single voxels with different director fields: along the edge (a), along the surface diagonal (b), and along the body diagonal (c); (i)-(v) represent the schematic director field (i), POM image of the voxels viewed from three different surfaces (ii), samples in (ii) rotated by 45° (iii), bight-field image of the voxels showing different surfaces (iv), samples in (iv) swelled with DMF (v). In (ii)-(v), F, R, and T corresponding to front surface, right surface, and top surface, respectively.

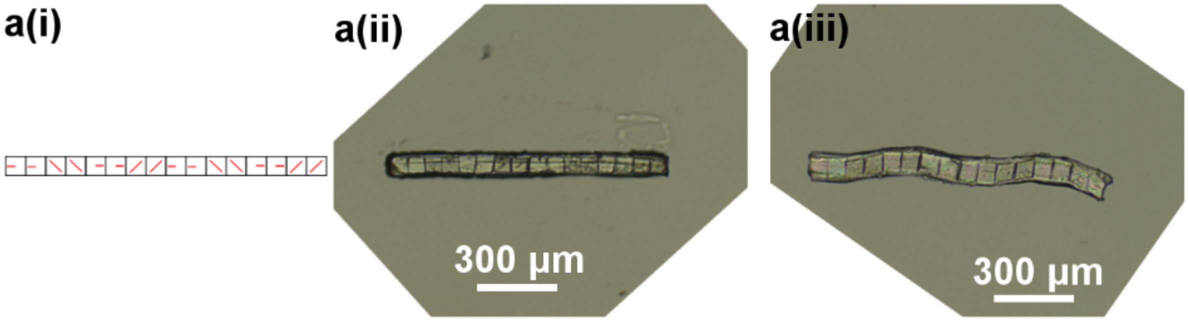

Supplementary Figure 3. **DMF swelling of an assembled 1D line.** i) Schematic of the programmed director field of the 1D line; ii) bright-field optical microscope image of the 1D line; iii) DMF swelling-based shape transformation of the 1D line.

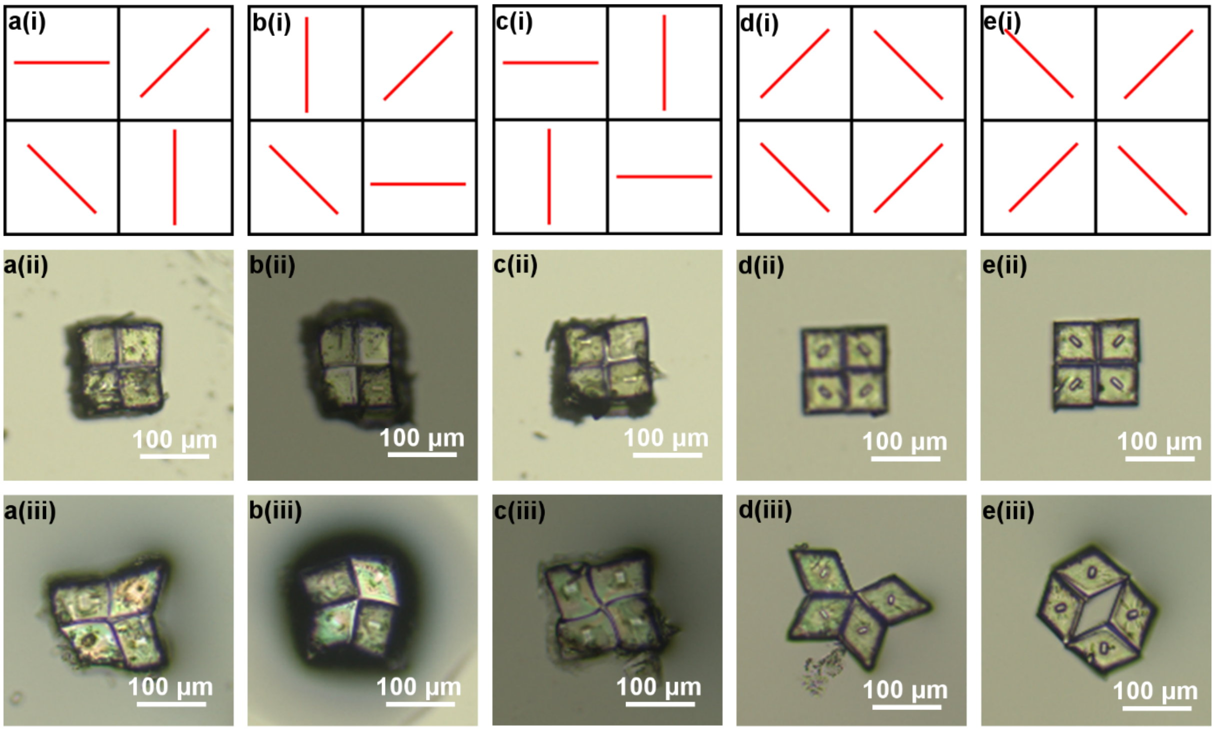

Supplementary Figure 4. **DMF swelling-based 2D-to-2D shape transformation of the assembled different 2D LCE structures.** a-c) Assembled 2D structures transforming into different shapes; (i)-(iii) represent schematic of the director fields (i), bright-field image of the assembled 2D structures (ii), and DMF swelling of the assembled 2D structures (iii). d, e) Assembled 2D structures with a non-glued surface transforming into different 2D shapes; (i)-(iii) represent schematic of the director fields (i), bright-field image of the assembled 2D structures (ii), and DMF swelling of the assembled 2D structures (iii).

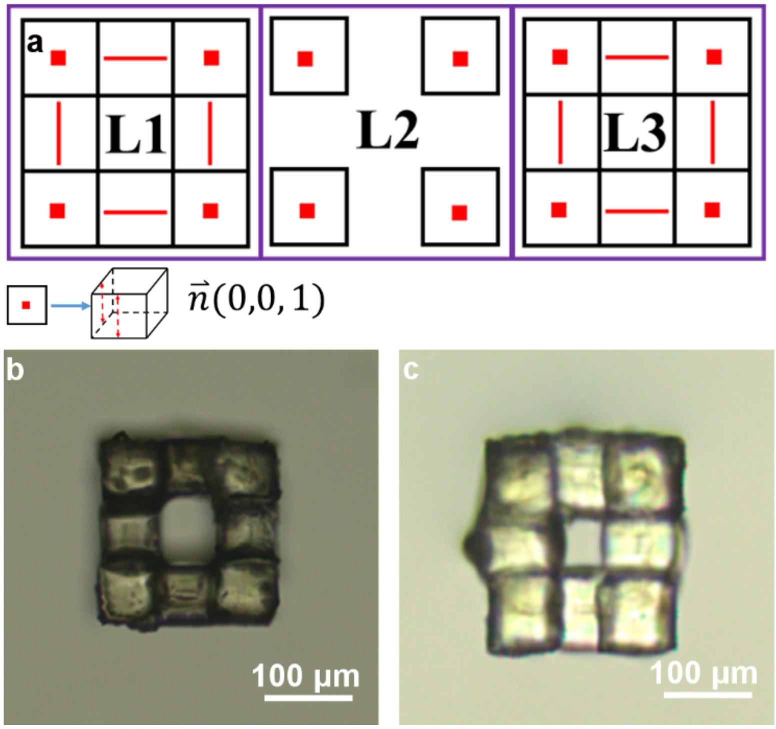

Supplementary Figure 5. **DMF swelling-based shape transformation of an assembled 3D LCE structure.** a) Schematic of the director field of a cuboid frame with 20 (three-layer) LCE voxels. b) Top-view bright-field optical microscope image of the assembled frame. c) The cuboid frame swelled with DMF.

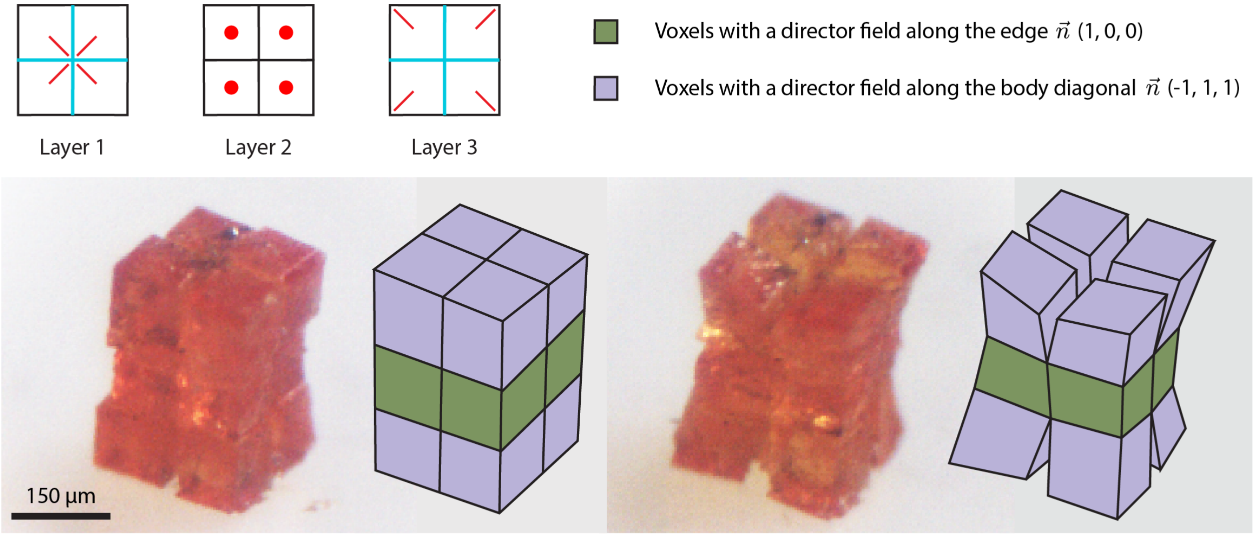

Supplementary Figure 6. **An LCE design with 100% fill factor.** The first row shows a schematic of the design, including both the geometry and the director field of 4 voxels in each layer. Two types of voxels, marked by green and purple color, with different director field orientations are used. The blue lines mark the contacts between adjacent voxels that are not glued together. The second row shows the actual experimental results before (left) and after (right) temperature-triggered shape transformation.

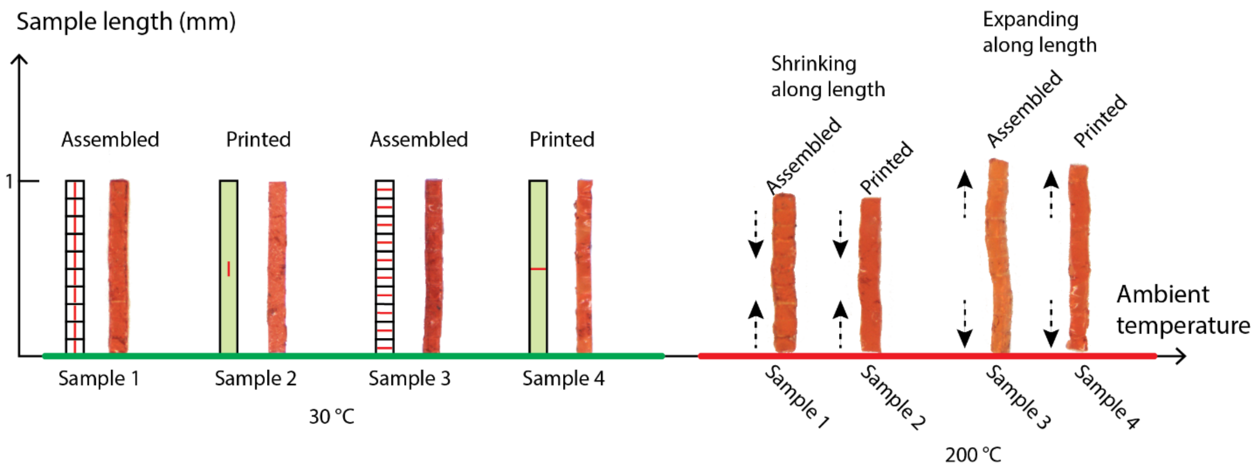

Supplementary Figure 7. **A comparison between four different LCE beams assembled by glue and the ones directly printed using two-photon polymerization.** The left side of the figure shows the designs and also the fabricated structures on a hotplate surface with a temperature of 30°C (green-colored horizontal line). Both vertical (Samples 1 and 2) and horizontal director fields (Samples 3 and 4) are investigated. The right side of the figure shows the shape transformation of the fabricated samples when the temperature of the hotplate surface (red-colored horizontal line) that the structures stay on is increased to 200 °C.
