# Supplementary material for: Shape-programmable liquid crystal elastomer structures with arbitrary three-dimensional director fields and geometries
Source: Nat Commun. 2021 Oct 12;12:5936. doi: 10.1038/s41467-021-26136-8 (PMC8511085; doi:10.1038/s41467-021-26136-8)
Supplement: Supplementary file 2 — Description of Additional Supplementary Files [file 41467_2021_26136_MOESM2_ESM.pdf]

Description of Additional Supplementary Files

File Name: Supplementary Movie 1
Description: The temperature-induced deformation is reversible for the structure shown in Fig. 5a.

File Name: Supplementary Movie 2
Description: UV illumination can cause a similar reversible deformation for the structure shown in Fig. 5b, with the help of dispersed red 1 acrylate
